# A *Plasmodium falciparum* Host-Targeting Motif Functions in Export during Blood Stage Infection of the Rodent Malarial Parasite *Plasmodium berghei*

**DOI:** 10.1371/journal.pone.0002405

**Published:** 2008-06-11

**Authors:** Julia J. MacKenzie, Noé D. Gómez, Souvik Bhattacharjee, Shaina Mann, Kasturi Haldar

**Affiliations:** Department of Pathology and Microbiology-Immunology, Northwestern University, Feinberg School of Medicine, Chicago, Illinois, United States of America; University of California Los Angeles, United States of America

## Abstract

*Plasmodium falciparum* (*P. falciparum*) secretes hundreds of proteins—including major virulence proteins—into the host erythrocyte. In order to reach the host cytoplasm, most *P. falciparum* proteins contain an N terminal host-targeting (HT) motif composed of 11 amino acids. *In silico* analyses have suggested that the HT motif is conserved throughout the *Plasmodium* species but experimental evidence only exists for *P. falciparum*. Here, we show that in the rodent malaria parasite *Plasmodium berghei* (*P. berghei*) a reporter-like green fluorescent protein expressed by the parasite can be exported to the erythrocyte cytoplasm in a HT-specific manner. This provides the first experimental proof that the HT motif can function as a signal for protein delivery to the erythrocyte across *Plasmodium* species. Further, it suggests that *P. berghei* may serve as a model for validation of *P. falciparum* secretome proteins. We also show that tubovesicular membranes extend from the vacuolar parasite into the erythrocyte cytoplasm and speculate that these structures may facilitate protein export to the erythrocyte.

## Introduction

Every year, several hundred million people are infected with *P. falciparum*, the cause of the most severe form of malaria in humans, and a significant fraction of infected individuals die from complications. All clinical ramifications of infection occur during the asexual blood-stage of infection.

During blood-stage infection, the parasite resides in a parasitophorous vacuole (PV) within the erythrocyte, a cell devoid of organelles, protein synthesis and trafficking machinery. Within this unusual host cell, the parasite matures intracellularly and directs elaborate host cell remodeling, including development of various membranous structures within the erythrocyte cytoplasm and the protrusion of the erythrocyte surface into so-called knobs.

These structural and antigenic changes are presumably required for both survival within the erythrocyte as well as avoidance of the host defenses [1]. A tubovesicular membrane network (TVN) that buds from the vacuolar parasite and extends to the erythrocyte membrane is needed for nutrient uptake [2]. Maurer's clefts, which are flattened, lamellar structures within the *P. falciparum-*infected erythrocyte have been linked to protein export from the PV to the erythrocyte cytoplasm and cell surface. Parasite proteins secreted from the parasite into the host erythrocyte presumably provide the molecular basis of host remodeling [1]. They are also thought to be responsible for important malaria disease pathologies. For instance, *P. falciparum* erythrocyte membrane protein-1 (*Pf*EMP1) and knob-associated histidine-rich protein (KAHRP) together form electron-dense protrusions on the surface of infected erythrocytes that are at least partially responsible for the highly virulent adhesive properties of *P. falciparum* [3]–[5].

In order to reach the host erythrocyte cytoplasm parasite proteins must traverse two membranes: the parasite plasma membrane and the PV membrane (PVM). A cleavable endoplasmic reticulum-like signal sequence (SS) is required to cross the parasite plasma membrane. For the majority of proteins, an N terminus host-targeting motif (HT) is required to cross the PVM into the host cytoplasm. The HT motif is an 11 amino acid sequence with a 5 amino acid core (RxLxE/D/Q) that is conserved between diverse proteins, collectively referred to as the “secretome” [6]. There are an estimated 300 to 400 proteins in the *P. falciparum* secretome [6]–[8].

*In silico* analyses have suggested that the HT motif is conserved throughout the *Plasmodium* species, including species of rodent maglaria parasites (RMP) such as *P. berghei, P. yoelii*, and *P. chabaudi* [6], [8], [9]. The secretome of RMPs is estimated to be significantly smaller than the *P. falciparum* secretome with no more than 60 proteins [6], [8]. The discrepancy may be due to several reasons, including incomplete annotation of RMP genomes. A second possibility may be reduced host-parasite interactions during RMP infection. In addition, a very small number of orthologues (∼10) are conserved across species [6]–[8]. Moreover parasite proteins that lack an HT motif have also been shown to be exported to the erythrocyte [10]. These analyses raise the question whether a signal present predominantly on hundreds of *P. falciparum*-specific gene products could efficiently target *P. berghei* proteins to the host erythrocyte.

All functional studies of the HT motif or closely related, but distinct, Plasmodium export element (PEXEL) have been done in *P. falciparum*. Previous studies using the N terminus of secreted *P. gallinaceum* (avian malaria parasite) and *P. vivax* (human malaria parasite) proteins transfected into *P. falciparum* demonstrate that their signaling motifs are recognized in *P. falciparum* [*9*]. However, there are no studies to date that investigate whether HT motifs are recognized in RMP. Here, we show that a *P. falciparum* HT motif expressed in *P. berghei* ANKA can target proteins to the host cytoplasm. Mutation of the HT motif abrogates export and mutated proteins are retained within the *P. berghei* PV. Further, we show that loss of the HT motif leads to accumulation of soluble protein cargo in the PV as well as tubular extensions emerging from the PV and extending into the erythrocyte. This suggests that the *P. berghei* vacuole is connected to tubovesicular membranes as detected in live cells and we speculate that these structures may confer species specific aspects of protein export to support host remodeling by RMPs.

## Results

### Integration of *P. falciparum* HRPII HT-GFP proteins into *P. berghei* ANKA by double crossover and expression of GFP transgenes

**Figure 1:**
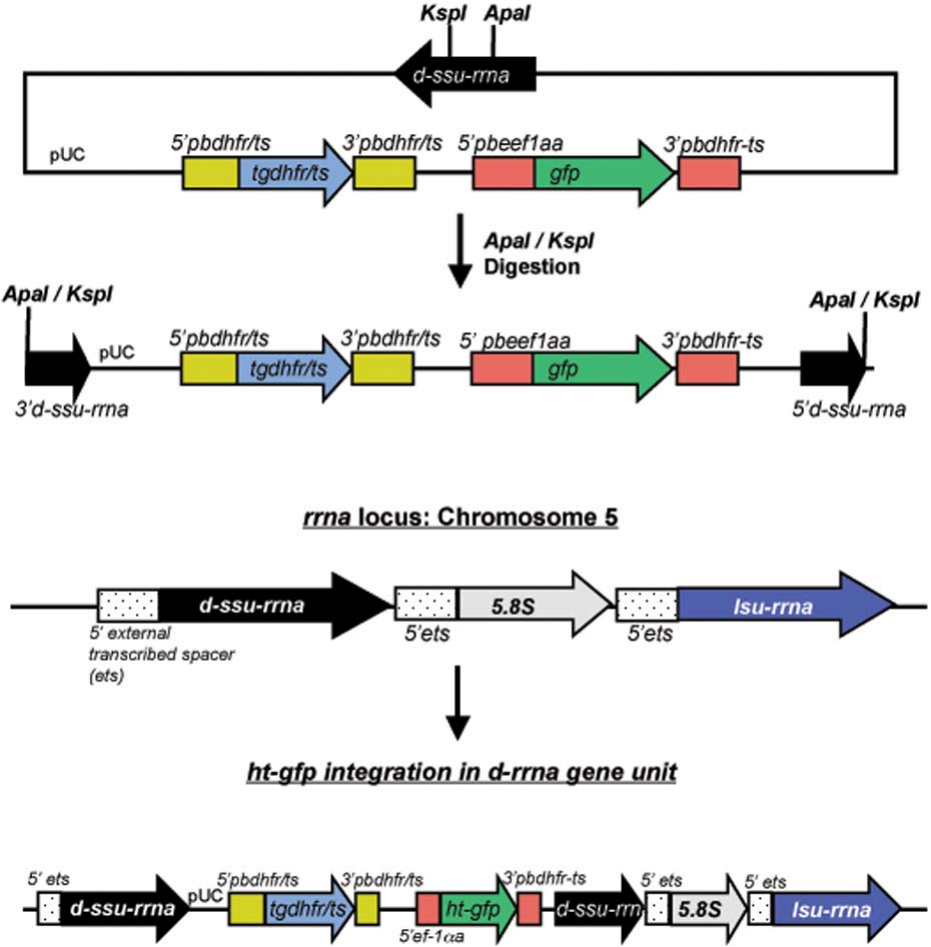
Strategy for integrating pL0016 (HT- or Δ-GFP) in *P. berghei* ANKA chromosome 5 by double crossover. The previously described double crossover replacement plasmid pL0016 (pPbGFP_CON_)[13] was used for cloning of HRPIIminhis.HT.GFP (a truncated HRPII gene containing a minimal histidine sequence, host-targeting motif, and GFP tag) and HRPIIminhis.Δ.GFP (a truncated HRPII gene containing a minimal histidine sequence, an 11 amino acid alanine-rich replacement of the host-targeting motif, and GFP tag).

To investigate HT motif recognition by *P. berghei*, we expressed a “minitransgene” containing the HT motif derived from a *P. falciparum* gene, *Pf*HRPII, fused to a green fluorescence protein (GFP) [11], [12] in a double crossover *P. berghei* replacement plasmid described in detail elsewhere, [13] (see methods and Figure 1). The full-length *Pf*HRPII protein contains an N-terminal SS and HT motif, followed by a sequence rich in histidine-repeats that extends to the C terminus. In contrast, the minitransgene contains the HRPII N terminus with either the wildtype HT motif (HRPIIminhis.HT.GFP) or an alanine-rich replacement HT motif (HRPIIminhis.Δ.GFP) followed by a single monomeric repeat of the histidine rich region fused to GFP. These truncated proteins were previously used to define minimal soluble reporters needed to detect and establish HT-mediated export of GFP in *P. falciparum* [6], [11], [12].

**Figure 2:**
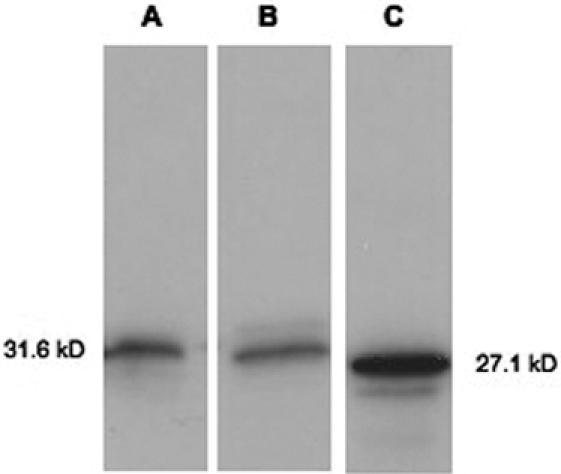
HT-GFP expression in *P. berghei-*HT infected BALB/C mouse whole blood lysates. Anti-GFP Western blots of (A) pL0016 (HT-GFP) (B) pL0016 (Δ-GFP) whole blood lysates and (C) recombinant GFP alone.

To test HT motif recognition in rodent malaria parasites, *P. berghei* ANKA schizonts were transfected with either HRPIIminhis.HT.GFP or HRPIIminhis.Δ.GFP using the Amaxa Nucleofector system and BALB/C mice were infected. Transfected parasites were selected for by pyrimethamine drug treatment. Expression of GFP in the blood stages of *P. berghei-*HT infection was confirmed by Western analysis. As shown in Fig. 2, a chimera of ∼31.6 kDa was detected in parasites expressing either HRPIIminhis.HT.GFP or HRPIIminhis.Δ.GFP and their migration in gels could be distinguished from that of recombinant, soluble GFP. These data suggest that the major green fluorescence signal associated with these infections are indeed chimeric GFPs of interest, rather than degradation products containing only free GFP.

### The *P. falciparum* HT motif is utilized in exporting GFP from the parasite to erythrocyte in *P. berghei* ANKA infections

**Figure 3:**
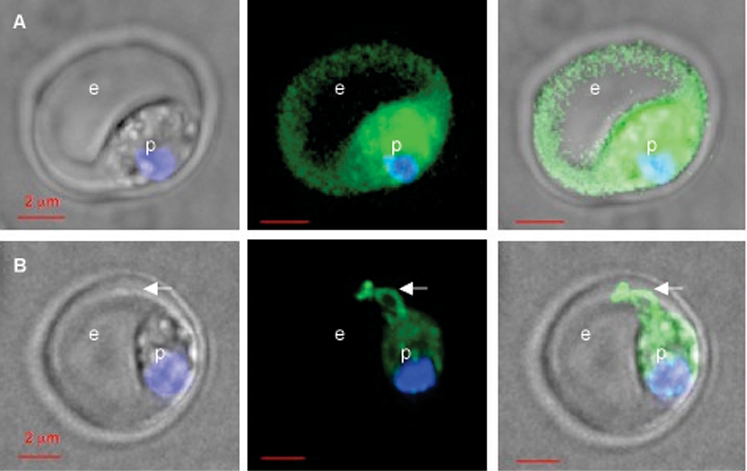
The HT motif of *P. falciparum* HRPII is recognized in *P. berghei* ANKA and transported to the erythrocyte cytoplasm. Export of the HT-containing N terminus of HRPII, a secretome protein specific to *P. falciparum*, depends on a functional HT motif when expressed in *P. berghei*. Live cell images of (A) HRPIIminhis.HT.GFP and (B) HRPIIminhis.Δ.GFP expression in *P. berghei-*infected erythrocytes isolated from infected mice. White arrow indicates tubovesicular structures extending from the PV to the periphery of the erythrocyte. e, erythrocyte, p, parasite.

The sub-cellular location of HRPIIminhis.HT.GFP and HRPIIminhis.Δ.GFP was investigated using live-cell microscopy. At a parasitemia of 17–30%, blood-stage parasites were collected from mice by cardiac puncture. As Fig. 3A shows, in parasitized erythrocytes obtained from mice infected with *P. berghei-*containing HRPIIminhis.HT.GFP, green fluorescence was readily detected in the host erythrocyte. Green fluorescence was also detected in the parasite, presumably because a constitutive promoter is used to drive transgene expression, resulting in ‘back up’ in the system. Replacement of the HT motif with an alanine-rich replacement sequence blocked release of GFP into the erythrocyte cytoplasm (Fig. 3B). Notably, GFP was detected in the body of the parasite as well as tubular extensions connected to vesicular elements at the periphery of the erythrocyte. These data definitively show that the HT motif can be utilized by *P. berghei* in exporting proteins to the erythrocyte cytoplasm and implies that the machinery for HT-dependent export is conserved throughout the genus *Plasmodium*. They also provide the first evidence of the presence of tubovesicular connections that extend between the vacuolar parasite and the periphery of the host erythrocyte, as detected in live cells.

## Discussion

Mouse models of malaria are important tools for studying malarial pathogenesis, yet they are limited by the differences between the genomes of *P. falciparum* and RMPs such as *P. berghei*. Based on *in silico* analyses, researchers have assumed that the HT motif present in *P. falciparum* secretome proteins is conserved in RMPs. There is limited information on RMP proteins exported to the erythrocyte, [14]. Here, we provide *in vivo* data that show that *P. berghei* recognizes the HT motif derived from *P. falciparum*. This provides definitive functional evidence for the use of this signal in *P. berghei* and suggests that the machinery necessary for the identification and processing of secreted malarial proteins is conserved between *P. falciparum* and *P. berghei*. Since genetic manipulation in *P. berghei* is more efficient than in *P. falciparum*, the data we present here suggest that evaluation of bioinformatics predictions of the *P. falciparum* secretome can be easily undertaken in *P. berghei*. This will enable much-needed comprehensive testing of predictive algorithms [6], [8], [9] and facilitate identification of putative malarial virulence effectors delivered to the host erythrocyte.

Our studies also provide the first live cell view of tubovesicular membrane organization induced in the host erythrocyte by *P. berghei*. This was unexpectedly revealed because these membranes are the site of reporter protein accumulation when the HT motif is replaced by an alanine-rich sequence. In *P. falciparum*, replacement of the HT motif primarily leads to a beaded protein accumulation pattern immediately around the parasite in a space consistent with the PV [6]. In addition, our recent studies show that in *P. falciparum*, the HT does not translocate protein across the PV. Rather, it enables protein sorting into Maurer's clefts that traverse from the parasite to the periphery of human erythrocytes and appear to be specialized secretory organelles utilized for protein export in these host cells [11]. Cleft organelles have not yet been reported in *P. berghei*, but it is possible that there are alternate *P. berghei* specialized structures that enable HT-mediated protein exit to the erythrocyte. Thus, *P. berghei* may possess features of erythrocytic remodeling both common to and distinct from *P. falciparum*. Further studies may reveal reasons for distinct *P. berghei*-specific organization of export structures, including inherent disparities in virulence of the two parasite species as well differences in size, membrane properties and circulatory functions between human and murine erythrocytes.

## Materials and Methods

### DNA constructs

The previously described double crossover replacement plasmid pL0016 (pPbGFP_CON_) [15]–[17] was used for cloning of **HRPIIminhis.HT.GFP** (a truncated HRPII gene containing a minimal histidine sequence, host-targeting motif, and GFP tag) and **HRPIIminhis.Δ.GFP** (a truncated HRPII gene containing a minimal histidine sequence, an 11 amino acid alanine-rich replacement of the host-targeting motif, and GFP tag), [12]. pL0016 contains a *Toxoplasma gondii* dihydrofolate reductase cassette (tgdhfr) for drug selection with perimethamine. We amplified HRPIIminhis.HT.GFP and HRPIIminhis.Δ.GFP from pBacII(HT-GFP^memb^myc) and pBacII(Δ-GFP^memb^myc), respectively, using the following primers: 5′ - ATAT **GGATCC** ATG GTT TCC TTC TCA AAA AAT AAA GTA TTA TCC – 3′ and 5′ - GGC C**GG ATC CC**T ATT TGT ATA GTT CAT CCA TGC CAT GTG TAA TCC C – 3′ (restriction sites marked in bold). pL0016 was digested with BamHI to remove GFP. PCR products for HRPIIminhis.WT.GFP and HRPIIminhis.Δ.GFP were digested with BamHI and ligated with pL0016. pL0016 (HT^sol^-GFP) and pL0016 (Δ^sol^-GFP) were digested with ApaI and SacII, and the expect 157 bp fallout was confirmed by electrophoresis. Digested DNA was precipitated in SET and ethanol, and resuspended in water for a final concentration of 1 µg/ml.

### Parasite cultures

Transformed parasites were generated as previously described [15]. Briefly, BALB/C mice (Charles River Laboratory) were infected with 1×10^6^wildtype *P. berghei* ANKA via intraperitoneal (IP) injection. At a parasitemia between 5–15%, 10 ul tail blood was collected in 0.4 ml PBS. The resulting suspension was immediately injected via IP injection into two mice. At day 4 post-injection with parasitemia between 2 and 4%, 1.5–2 ml infected blood from two infected mice was collected by cardiac puncture under anesthesia between 13:00 and 15:00. Blood was added to 5 ml complete RPMI1640 media supplemented with 0.3 ml heparin. Infected erythrocytes were harvested, resuspended in 50 ml complete media, and distributed equally between two culture flasks. Flasks were flushed with a gas mixture (5% CO_2_, 5% O_2_, 90% N_2_) using a 0.2 µm filter unit for 90 seconds, and incubated between 36°C–37°C until 09:00 the next morning.

Following overnight culture, a thin blood smear was made, fixed, stained with geimsa, and viewed with immersion oil and ×100 objective. It was verified that 70–80% of parasites were morphologically viable mature schizonts. To purify schizonts, 100 ml culture suspensions were distributed among three 50 ml tubes. 10 ml of 50% Nycodenz-PBS was underlayed in each tube. Tubes were centrifuged without brake for 30 min at 450 g. Schizonts were collected from the interface between the two resulting suspensions and washed in complete media. Schizonts were carefully resuspended in 10 ml complete media and distributed among ten eppendorf tubes. Each tube was then used in separate transfections.

### Transfection and drug selection of genetically transformed *P. berghei*

Mice were injected with 150 µl of 6 mg/ml phenylhydrazine HCl 3 days before the day of transfection. Schizonts were transfected using the Amaxa Nucleofector system (Amaxa Biosystems), as previously described [15]. Briefly, schizonts were pelleted by centrifugation and supernatants were discarded. 100 ul of 88A6 Nucleofector solution containing 5–10 ul of DNA was added to resuspend schizonts. Schizonts and DNA in buffer solution were transferred to an electroporation cuvette and schizonts were transfected using the Amaxa gene pulser and program U33 (Amaxa Biosystems). 50 ul of complete culture medium was immediately added to pulsed samples and the total solution was transferred to an Eppendorf tube.

Using an insulin syringe, the complete transfection solution (150 ul) was injected into a tail vein of a mouse under anesthesia. Drinking water containing pyrimethamine (70 ug/ml) was provided to the mice one day after transfected parasites had been injected. Pyrimethamine-containing water was provided for a 4 day period. 4 days after removing drug pressure, thin blood smears from tail blood were made every day to monitor parasitemia. Parasites typically came up 6–10 days post-transfection. At a parasitemia of 2–5%, infected erythrocytes were passaged into a new mouse and a second round of drug pressure was applied immediately, for 4 days.

At a parasitemia of 2–5%, one droplet of tail blood was collected in 200 ul PBS. 4 ul of Hoechst 33258 was added and incubated for 5 minutes. 5 ul of the resulting solution was suspended on a microscope slide under a cover slip, and GFP fluorescence was confirmed using a fluorescent microscope (objective at ×40).

### Preparation of lysates for SDS-PAGE and Western blots

Whole blood was lysed with sterile water and immediately boiled in 2× SDS sample buffer for 5 minutes. Lysates and recombinant HRPIIminhis.WT.GFP, HRPIIminhis.Δ.GFP and GFP proteins were separated by SDS-PAGE, and transferred to PVDF membranes. The membranes were incubated with 5% nonfat dry milk in PBS containing 0.05% Tween for 30 minutes and subsequently incubated with rabbit anti-GFP antibody (1∶ 2000) for 1 h at room temperature. Membranes were then washed with PBS/Tween and incubated with peroxidase-conjugated goat anti-rabbit secondary antibody (1∶ 2500) for 1 h at room temperature. The bands were developed with ECL solutions (Pierce) using the manufacturer's instructions.

### Isolation of infected erythrocytes and live cell microscopy

Under anesthesia (Nembutal with an i.p dose of 50 mg/kg of body weight), blood was collected from mice via cardiac puncture using a 25-gauge needle. Parasitemia of mice at the time of cardiac puncture ranged from 17–30%. Blood was centrifuged at 2000 rpm for 5 minutes to separate whole blood from plasma. Separate stocks were made of each, and frozen at −80°C.

For live cell microscopy, isolated whole blood was immediately stained with 10 ug/ml Hoechst for 5 min. To visualize transfected parasites expressing GFP, fluorescence microscopy and digital-image collection were performed on an Olympus (New Hyde Park, NY) IX inverted fluorescence microscope and Photometrix (Tucson, AZ) cooled charge-coupled device camera (CH350/LCCD) driven by DeltaVision software from Applied Precision Inc. (Seattle, WA). Twenty optical sections spaced 200 nm apart were taken through the depth of the cells using a 100× objective.

### Ethical Treatment of Research Subjects

This research was approved by the Northwestern University Animal Care and Use Committee (NUACUC) under protocol number 2007-1155.
